# Association of main meal quality index with the odds of metabolic syndrome in Iranian adults: a cross-sectional study

**DOI:** 10.1186/s40795-023-00711-2

**Published:** 2023-03-21

**Authors:** Amin Mirrafiei, Mohaddeseh Hasanzadeh, Fatemeh Sheikhhossein, Maryam Majdi¹, Kurosh Djafarian, Sakineh Shab-Bidar

**Affiliations:** 1 Department of Community Nutrition, School of Nutritional Sciences and Dietetics, Tehran University of Medical Sciences (TUMS), 14167-53955 Tehran, Iran; 2 Nutritional Health Team (NHT), Universal Scientific Education and Research Network (USERN), Tehran, Iran; 3 Department of Clinical Nutrition, School of Nutritional Sciences and Dietetics, Tehran University of Medical Sciences (TUMS), 14167-53955 Tehran, Iran; 4 Department of Community Nutrition, School of Nutritional Sciences and Dietetics, Tehran University of Medical Sciences (TUMS), No 44, Hojjat-dost Alley, Naderi St., Keshavarz Blvd, P. O. Box 14155/6117, Tehran, Iran

**Keywords:** Meal, Diet quality, Metabolic syndrome, Main meal quality index

## Abstract

**Background:**

Metabolic syndrome (MetS) is a common global issue linked to the quality of one’s eating occasions. The current cross-sectional study evaluates the association between a novel index, the Main Meal Quality Index (MMQI), and MetS among Iranian adults.

**Methods:**

A total of 824 men and women were recruited, and a 24-hour dietary recall assessed the dietary intake of the participants. Lunch was selected as the main meal based on energy density. The MMQI score was calculated based on ten components of dietary intake, with a higher score indicating more adherence to the index, with the final scores ranging from 0 to 100 points. The associations were assessed using binary logistic regression.

**Results:**

The mean age was 42.2 years and the range of the calculated MMQI was 22 to 86 (mean in total participants: 56.62, mean in women: 56.82, mean in men: 55.64). The total prevalence of MetS in the sample was 34%. After adjustments for potential confounders, the participants at the top quartile of MMQI had a lower odds ratio for hypertriglyceridemia and low high-density lipoprotein (HDL) level, and a higher odds ratio for hypertension, hyperglycemia, abdominal obesity, and MetS. The sex-specific analysis also did not show any significant associations between adherence to MMQI and MetS and its components.

**Conclusion:**

Overall, MMQI is not associated with MetS and its components in a sample of Iranian men and women. More research is needed to examine MMQI and its possible association with current health-related problems including MetS.

## Introduction

Metabolic syndrome (MetS) is defined as a bundle of metabolic abnormalities consisting of abdominal obesity, impaired glucose tolerance, high blood pressure, an upraised triglyceride (TG) level, and a lessened high-density lipoprotein cholesterol (HDL-C) level (1) that increases the risk of type 2 diabetes (T2D) fivefold (2) and doubles the chance of cardiovascular disease (CVD) (3). Diet, as a key part of behavior adjustment, is beneficial for all the components of MetS (4, 5), such as dyslipidemia (6), hypertension (7), adiposity (8), insulin resistance (9), and hyperglycemia (10, 11), mainly by contributing to the depletion of additional weight (5). To interpret this more precisely, the risk of MetS, is predicted by indicators of diet quality that are utilized in research worldwide. Because there are so many different guidelines and requirements for the consumption of various nutrients, it is challenging to define clearly what constitutes a high-quality diet (12).

Based upon multiple types of research and estimations over time, several indices have been introduced to evaluate overall diet quality (13, 14). These indices assess particular dietary patterns, such as the dietary inflammatory index (DII) (15), or guidelines presented at a regional level (16). Among demographic and cultural differences, nutritional scores and indices have been developed for global populations, independent of social and racial circumstances. These scores aim to enhance the quality of eating when facing a specific condition or as a habitual diet guideline (17, 18). Most of these recommendations have some components in common, for instance increasing the daily consumption of vegetables, fruits, fibers, and whole grains, and reducing the intake of saturated fats, processed foods, and sugary products (19). On the other hand, focusing on the meals consumed by people might be a more straightforward method for understanding the significance of healthy intake in the prevention of chronic diseases, as has been shown through different studies on this particular issue (20–22).

This led to the development of a novel indicator for assessing diet quality named the Main Meal Quality Index (MMQI), which is specifically designed for the main meal of the day (23), as dietary guidelines based on meals may be a useful tool in aiding people to maintain a healthy lifestyle due to their easy-to-understand and comprehensive nature (24, 25). In addition, a single meal could be sufficient to promote health benefits (26).

No previous study has evaluated the association between meal quality and MetS in Iranian adults. Due to the high prevalence of MetS in Iranians (30.4%) (27), this study aims to inspect the association between MMQI (in this case, lunch, as the main contributor to the total daily energy intake) and the probability of MetS and its components in Iranian adults for the first time.

## Subjects and methods

### Study design and participants

The current cross-sectional study was conducted on 824 healthy adults attending health centers affiliated with Tehran University of Medical Sciences in 2017–2018 who were sorted out by a multi-stage cluster random sampling method from the 5 regions (north, south, west, east, and center) of Tehran. Multiple health centers from each region were chosen, and qualified individuals from each center were selected by the mean of easy sampling, based on the proportion of the total number of people referring to a health center. The study sample size of 850 was calculated employing the formula: n = (pqz²)/E², contemplating that n = sample size; z² = square of the confidence level in standard error units (1.96); p = the estimate of the proportion of healthy adults; q = 1 − p, or the estimated proportion of people with metabolic syndrome; and E² = the square of the maximum allowance for error between the true proportion and the sample proportion (0.04) (28, 29). The inclusion criteria were adults in the age range of 20 to 60 years, apparently healthy, eager to participate in the study, a member of a health center, and a resident of Tehran. They were informed about the purpose of the study and filled out the consent to participate in it. The exclusion criteria consisted of a history of diabetes, cancer, and CVD, a possible change to the usual diet before participation, and lactating and pregnant women. A demographic questionnaire consisting of age, sex, education, marital status, occupation, and smoking status was used by experienced interviewers to gather and record general information about the subjects.

### Anthropometric measures and blood pressure

We used a stadiometer with a sensitivity of 0.1 cm (Seca, Hamburg, Germany) and a digital scale instrument with a precision of 0.1 kg (808Seca; Seca) to evaluate anthropometric measures compromising body height and weight. Participants were dressed in light clothing without shoes. Body Mass Index (BMI) was calculated individually and participants were divided into four categories of underweight (< 18.5), normal (≤ 18.5–24.9≥), overweight (≤ 25-29.9≥), and obese (≥ 30) (30). Waist and hip circumferences were measured between the iliac crest and lower ribs by a flexible measuring tape. Physical activity was evaluated using a validated International Physical Activity Questionnaire (IPAQ) (31). Systolic and diastolic blood pressure were evaluated in a sitting stance by a digital sphygmomanometer (BC 08; Beurer, Ulm, Germany) after a resting time of ten to fifteen minutes. Blood pressure was measured twice in each participant and the average amount was entered into the analysis.

### Dietary assessment

The dietary intake of the participants was recorded using a 24-hour recall questionnaire on three non-consecutive days. A trained dietician collected the first recall via a face-to-face interview, and the next two recalls were gathered by phone calls on random days of the week. Meals, energy, and food groups were determined by the dietary recalls, and micro and macronutrients were extracted utilizing the Nutritionist IV software.

### Meal definitions

Meals were known as occasions where large amounts of food were consumed or were standardized based on time of consumption (32, 33) to contain no more than one breakfast, lunch, and dinner, but allow for multiple snacks. Based on prior studies, breakfast was defined as an eating occasion where a large amount of food or energy was consumed between 5:00 and 11:00; lunch, if it was consumed between 11:00 and 16:00; and dinner, if it was eaten between 16:00 and 23:00 (34).

### Calculating MMQI

The main meal of the day, lunch, was selected based on its contribution to total calorie intake, and the MMQI was evaluated by the standards stated by Gorgulho et al. The components and scoring system are expressed in Table 1 (23). The MMQI is based on 10 components: fruit, vegetables (except potatoes), animal protein/total protein ratio, fiber, carbohydrates, total fat, saturated fat, processed meat, sugary beverages and desserts, and energy density. A score range of 0 to 10 points is clarified for every single component; thus, the final score varies between 0 and 100 points for each individual. To get the maximum score, an individual must consume at least 80 grams of fruit and 160 grams of vegetables during the main meal. More than 20% of the protein intake must come from plant sources, and a minimum of 10 grams of dietary fiber should be consumed. Based on the WHO recommendation, total carbohydrates ought to supply above 55% of total energy intake (maximum 75%), total fat below 30% of total energy intake (minimum 15%), and saturated fat lower than 10% of total energy intake. Complete avoidance of sugary beverages, desserts, and processed meats is considered optimal, and lastly, an energy density of less than 1.25 kcal/g is applied for a perfect MMQI score. The correlation between MMQI and determined nutrient intake of the lunch meal was assessed using univariate linear regression, adjusting for age and sex.

**Table 1:** Mean MMQI scores and distribution of adults in MMQI categories according to socioeconomic, demographic and anthropometric characteristics

Characteristics	Mean	95% CI	P	Total population (n)	1st tertile (n)	2nd tertile (n)	3rd tertile (n)	*P value
Overall population	56.62	55.85–57.37	-	824	275	275	274	-
Sex								
Men	55.59	53.74–57.45	0.231	142	48	49	45	0.90
Women	56.82	55.99–57.66		682	227	226	229	
Body weight status								
Underweight	49.90	45.42–54.38	0.167	10	7	2	1	0.19
Normal	56.49	55.14–57.83		270	95	85	90	
Overweight	56.37	55.18–57.56		332	111	114	107	
Obese	57.46	55.94–58.99		212	62	74	76	
Education								
Illiterate	57.93	54.96–60.90	0.346	55	15	16	24	0.66
Under-diploma	56.90	55.35–58.46		195	65	65	65	
Diploma	57.07	55.73–58.41		286	93	95	98	
Academic	55.70	54.44–56.96		288	102	99	87	
Occupation								
Employed	55.99	54.73–57.26	0.295	302	104	103	95	0.81
Housekeeper	57.24	56.20-58.28		431	139	143	149	
Retired	56.91	53.48–60.35		47	13	17	17	
Unemployed	54.63	50.98–58.28		43	18	12	13	
Marital status								
Single	55.54	53.42–57.66	0.540	111	40	38	33	0.96
Married	56.88	56.04–57.72		664	218	222	224	
Divorced	56.50	48.21–64.79		12	4	3	5	
Widowed	55.03	50.89–59.16		37	13	12	12	
Smoking								
Non-smokers	56.58	55.80-57.35	0.654	796	266	266	284	0.74
Smokers	57.54	53.56–61.52		28	9	9	10	

*Mean and 95% confidence intervals (95% CI) are described and p-value between groups using ANOVA. Number of participants in each category of MMQI were evaluated using Pearson’s chi-square.

### Laboratory investigations

Each participant provided a 12-hour fasting blood sample for the quantification of fasting plasma glucose (FPG), TG, and HDL. Blood samples were measured by standard methods at the Nutrition and Biochemistry Laboratory of the School of Nutritional Sciences and Dietetics at Tehran University of Medical Sciences. Glucose was assayed by the enzymatic (glucose oxidase) colorimetric method. Commercial kit (Pars Azmoon, Tehran, Iran). Serum total cholesterol (TC) and high-density lipoprotein-cholesterol (HDL-C) were measured using a cholesterol oxidase phenol amino antipyrine method, and triglyceride (TG) was measured using a glycerol-3 phosphate oxidase phenol amino antipyrine enzymatic method. All these tests were done by commercial kits (all from Pars Azmoon, Iran) using an auto-analyzer system (Selectra E, Vitalab, the Netherlands).

### Metabolic syndrome definition

We used the criteria of the National Cholesterol Education Program Adult Treatment Panel III (NCEP ATP III) to define MetS. As per the guideline, MetS is explained as the presence of three or more of the following criteria: 1) increased waist circumference (WC) (> 102 cm [> 40 in] for men, > 88 cm [> 35 in] for women); 2) elevated TG (≥ 150 mg/dl); 3) low HDL cholesterol (< 40 mg/dl in men, < 50 mg/dl in women); 4) hypertension (≥ 130/≥85 mmHg); and 5) impaired fasting glucose (≥ 110 mg/dl) (35).

### Statistical analysis

We utilized the Statistical Package for the Social Sciences (SPSS version 26; SPSS Inc., Chicago, IL, USA) to carry out all of the statistical analyses. The p < 0.05 was considered significant. We used a one-way analysis of variance test (ANOVA) to compute the mean of the participants for every component of the MMQI, and the association between MMQI and nutrient intake was assessed by linear regression, adjusting for age and sex. Subsequently, the MMQI score was divided into tertiles and the general characteristics of subjects among tertiles of the index score were compared using Chi-square for qualitative variables and the ANOVA test for continuous variables. The mean level of the biochemical parameters was also compared across tertiles of MMQI using analysis of covariance (ANCOVA). We used binary logistic regression to assess the relationship between MMQI and the likelihood of MetS in the crude Model, Model 1, adjusted for education, occupation, marital status, smoking status, and activity score, and Model 2, additionally adjusted for sex, age, and mean energy intake. The first tertile of the MMQI was considered the reference category. Due to the sex differences in etiology, biology, and clinical expression of MetS, we conducted the analyses on men and women separately.

## Results

**Figure 1:**
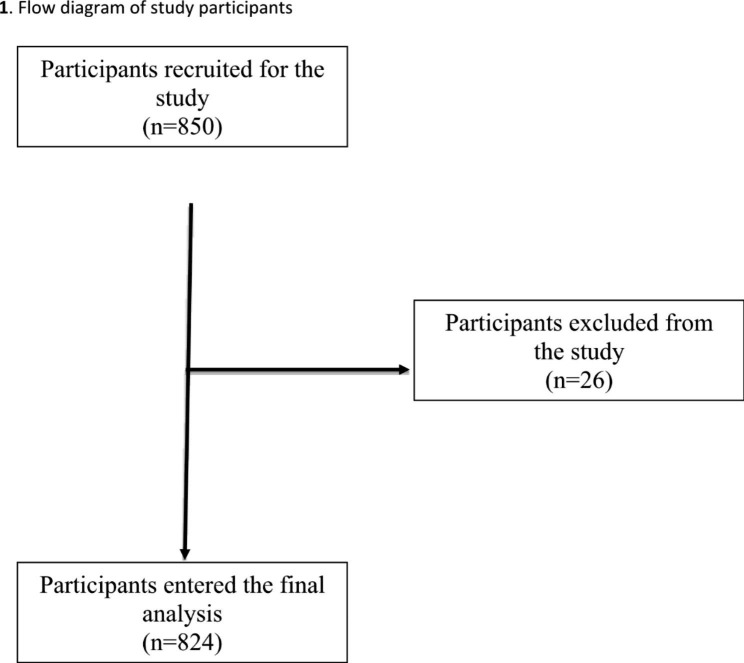
Flow diagram of study participants

Twenty-six participants dropped out due to under or over-reporting of energy intake, and the final sample size of 824 people entered the concluding analysis (Fig. 1). The majority of the sample size were women, non-smokers, the married, housekeepers, with an academic degree. The mean age of the participants was 42.2 ± 10.5 years. Table 1 shows the general characteristics of participants across MMQI categories. Women had a higher average score of MMQI compared to men (P = 0.23). Also, obese participants (P = 0.17), smokers (P = 0.65), married participants (P = 0.54), illiterates (P = 0.35), and housekeepers (P = 0.29) possessed a higher mean MMQI score than others in their subgroup, but none of them were significant.

The standard for scoring and the mean of each component of the MMQI in the study population is presented in Table 2. The 25th, 50th, and 75th percentiles of MMQI score for every dietary component are also shown in Table 2.

**Table 2:** Main Meal Quality Index components, standards for scoring and average values

Components	Standard for maximum score (10)	Standard for minimum score (0)	Mean	SEM	Lower CI	Upper CI	P 25	P 50	P 75
Fruit	≥ 80	0	0.55	0.034	0.49	0.62	0.00	0.00	1.00
Vegetable (excluded potato)	≥ 160	≤ 80	0.96	0.076	0.82	1.10	0.00	0.00	0.00
Animal protein/ total protein	≤ 80%	100%	8.63	0.115	8.39	8.87	10.00	10.00	10.00
Fiber	≥ 10	≤ 7	2.76	0.137	2.48	3.06	0.00	0.00	6.00
Carbohydrate	≥ 55% of total energy	≤ 40 of total energy	5.71	0.124	5.46	5.93	3.00	6.00	9.00
Total fat	≤ 30% of total energy	≥ 40% of total energy	5.57	0.139	5.28	5.84	1.00	6.00	10.00
Saturated fat	≤ 10% of total energy	≥ 13% of total energy	8.43	0.115	8.20	8.65	10.00	10.00	10.00
Processed meat	0 portion	1 portion (190 kcal)	9.70	0.045	9.61	9.78	10.00	10.00	10.00
Sugary beverages and desserts	0 portion	1 portion (110 kcal)	9.47	0.046	9.37	9.55	10.00	10.00	10.00
Energy density	≤ 1.25 kcal/ gr	≥ 1.65 kcal/gr	4.83	0.158	4.53	5.13	0.00	5.00	10.00
Final score	-	-	56.62	0.388	55.85	57.37	49.00	57.00	65.00

Abbreviations: SEM, standard error of the mean

Results of Table 3 shows that the final score was positively associated with carbohydrates (P < 0.001), calcium (P = 0.01), vitamin A (P = 0.04), and vitamin C (P < 0.001), and was negatively associated with the energy (P < 0.001), protein (P < 0.001), total fat (P < 0.001), saturated fat (P < 0.001), cholesterol (P < 0.001), polyunsaturated fat (PUFA) (P < 0.001), monounsaturated fat (MUFA) (P < 0.001), zinc (P < 0.001) and sodium (P = 0.01).

**Table 3:** Association between MMQI and nutrient intake

Nutrients	β	SE	95% CI	*P value
Energy (kcal)	-2.52	0.53	-3.56 to -1.47	< 0.001
Carbohydrate (g)	0.40	0.07	0.26 to 0.54	< 0.001
Protein (g)	-0.11	0.02	-0.15 to -0.06	< 0.001
Total fat (g)	-0.41	0.02	-0.46 to -0.36	< 0.001
Saturated fat (g)	-0.11	0.01	-0.13 to -0.09	< 0.001
Cholesterol (mg)	-1.17	0.19	-1.54 to -0.80	< 0.001
Polyunsaturated fat (g)	-0.09	0.01	-0.11 to -0.07	< 0.001
Monounsaturated fat (g)	-0.13	0.02	-0.18 to -0.09	< 0.001
Zinc (mg)	-0.01	0.003	-0.02 to -0.008	< 0.001
Selenium (mcg)	0.00	0.006	-0.01 to 0.01	0.99
Iron (mg)	0.02	0.02	-0.03 to 0.07	0.43
Calcium (mg)	1.18	0.37	0.46 to 1.90	0.01
Sodium (mg)	-4.14	1.24	-6.57 to -1.70	0.01
Vitamin A (RE)	1.59	0.79	0.30 to 3.14	0.04
Vitamin E (mg)	-0.02	-0.01	-0.03 to 0.001	0.07
Vitamin C (mg)	0.30	0.07	0.15 to 0.44	< 0.001

Abbreviations: CI, confidence interval; MMQI, Main Meal Quality Index. Linear regression between MMQI and each nutrient adjusted by gender and age

*obtained by linear regression analysis

Metabolic biomarkers of the study participants across the tertiles of the MMQI are presented in Table 4. After adjusting for sex, age, occupation, marriage, smoking, energy intake, physical activity, and education, subjects in the highest quartile had a higher value of TC, LDL, HDL, SBP, DBP, and BMI. Also, compared with the participants in the first tertile of the MMQI, those in the top quartile had a lower value of FPG and TG, while WC was the same value between the first and the last tertile, although only DBP showed statistically significant results (P = 0.03).

**Table 4:** Metabolic biomarkers of participant according to MMQI tertile

	1st tertile	2nd tertile	3rd tertile	*P value
FPG (mg/dl)	108.3 ± 2.12	106.8 ± 2.12	108.1 ± 2.13	0.86
TG (mg/dl)	150.1 ± 4.62	137.2 ± 4.61	146.9 ± 4.64	0.12
TC (mg/dl)	195.0 ± 2.64	194.0 ± 2.63	198.4 ± 2.65	0.46
LDL (mg/dl)	115.1 ± 2.27	116.8 ± 2.27	118.8 ± 2.28	0.51
HDL (mg/dl)	49.8 ± 0.60	49.7 ± 0.60	50.2 ± 0.60	0.84
SBP (mmHg)	118.0 ± 0.87	116.9 ± 0.87	119.4 ± 0.87	0.14
DBP (mmHg)	79.5 ± 0.57	77.6 ± 0.57	79.2 ± 0.57	0.03
WC (cm)	89.3 ± 0.66	89.0 ± 0.66	89.3 ± 0.66	0.93
BMI (kg/m²)	27.0 ± 0.26	27.4 ± 0.26	27.3 ± 0.26	0.54

FPG Fasting Plasma Glucose, TG triglyceride, TC total cholesterol, LDL low density lipoprotein, HDL high density lipoprotein, SBP systolic blood pressure, DBP diastolic blood pressure, WC waist circumference, BMI body mass index

Values are based on mean ± standard error

*adjusted for sex, age, occupation, marriage, smoking, energy intake, physical activity, and education

Multivariate adjusted odds ratios and 95% confidence intervals for metabolic syndrome and its components across tertiles of MMQI in the total population, women, and men are provided in Table 5. In the total population, those who were in the highest tertile of the MMQI were more likely to have MetS (OR: 1.04; 95% CI: 0.70,1.54; P = 0.86), hypertension (OR: 1.04; 95% CI: 0.63,1.69; P = 0.91), hyperglycemia (OR: 1.15; 95% CI: 0.81,1.64; P = 0.42), and greater abdominal obesity (OR: 1.14; 95% CI: 0.72,1.79; P = 0.58), and a lower risk of hypertriglyceridemia (OR: 0.92; 95% CI: 0.61,1.25; P = 0.43) and low HDL-C level (OR: 0.97; 95% CI: 0.68,1.37; P = 0.86), compared with participants in the lowest tertile of MMQI after adjustment of possible confounders. In the sex subgroups, women in the highest tertile had a lower OR for hypertriglyceridemia (OR: 0.88; 95% CI: 0.60,1.13; P = 0.50) and low HDL (OR: 0.92; 95% CI: 0.64,1.34; P = 0.70), and a higher OR for MetS (OR: 1.19; 95% CI: 0.79,1.78; P = 0.41), hypertension (OR: 1.06; 95% CI: 0.63,1.80; P = 0.87), abdominal obesity (OR: 1.36; 95% CI: 0.92,2.01; P = 0.14), and hyperglycemia (OR: 1.15; 95% CI: 0.78,1.68; P = 0.49) in the fully adjusted model. In men, high adherence to MMQI was associated with a lower chance of MetS (OR: 0.43; 95% CI: 0.14, 1.39; P = 0.18) and all of its components. None of these associations in Table 5 were statistically significant in any model.

**Table 5:** Multivariate adjusted odds ratios and 95% confidence intervals for metabolic syndrome and its components across tertiles of MMQI

	1st tertile	2nd tertile	3rd tertile	P trend*
Hypertriglyceridemia (total)				
Crude	1.00	0.76 (0.54,1.07)	0.85 (0.60,1.20)	0.34
Model 1	1.00	0.71 (0.50,1.02)	0.86 (0.60,1.23)	0.39
Model 2	1.00	0.70 (0.49,1.00)	0.92 (0.61,1.25)	0.43
Hypertriglyceridemia (women)				
Crude	1.00	0.84 (0.57,1.24)	0.85 (0.58,1.25)	0.40
Model 1	1.00	0.81 (0.54,1.19)	0.87 (0.59,1.28)	0.47
Model 2	1.00	0.80 (0.53,1.18)	0.88 (0.60,1.13)	0.50
Hypertriglyceridemia (men)				
Crude	1.00	0.64 (0.29,1.42)	0.96 (0.42,2.20)	0.91
Model 1	1.00	0.63 (0.28,1.41)	0.95 (0.41,2.19)	0.90
Model 2	1.00	0.60 (0.26,1.38)	0.96 (0.42,2.23)	0.92
Hypertension (total)				
Crude	1.00	0.64 (0.39,1.05)	1.00 (0.64,1.58)	0.98
Model 1	1.00	0.56 (0.33,0.95)	1.07 (0.66,1.74)	0.80
Model 2	1.00	0.56 (0.33,0.95)	1.04 (0.63,1.69)	0.91
Hypertension (women)				
Crude	1.00	0.65 (0.37,1.12)	1.01 (0.62,1.66)	0.98
Model 1	1.00	0.58 (0.33,1.02)	1.10 (0.65,1.86)	0.76
Model 2	1.00	0.57 (0.32,1.02)	1.06 (0.63,1.80)	0.87
Hypertension (men)				
Crude	1.00	0.66 (0.21,2.08)	0.77 (0.24,2.43)	0.64
Model 1	1.00	0.56 (0.17,1.85)	0.64 (0.19,2.14)	0.46
Model 2	1.00	0.61 (0.18,2.06)	0.64 (0.19,2.18)	0.48
Hyperglycemia (total)				
Crude	1.00	1.06 (0.76,1.48)	1.13 (0.81,1.58)	0.46
Model 1	1.00	1.02 (0.72,1.44)	1.17 (0.83,1.67)	0.36
Model 2	1.00	1.02 (0.72,1.45)	1.15 (0.81,1.64)	0.42
Hyperglycemia (women)				
Crude	1.00	0.99 (0.68,1.43)	1.12 (0.78,1.62)	0.54
Model 1	1.00	0.94 (0.64,1.38)	1.17 (0.80,1.71)	0.42
Model 2	1.00	0.94 (0.64,1.38)	1.15 (0.78,1.68)	0.49
Hyperglycemia (men)				
Crude	1.00	1.15 (0.52,2.56)	0.85 (0.37,1.96)	0.71
Model 1	1.00	1.33 (0.58,3.07)	0.94 (0.39,2.25)	0.91
Model 2	1.00	1.44 (0.60,3.41)	0.94 (0.39,2.28)	0.91
Low HDL-C (total)				
Crude	1.00	1.23 (0.88,1.71)	0.92 (0.66,1.29)	0.63
Model 1	1.00	1.26 (0.89,1.78)	0.97 (0.69,1.37)	0.87
Model 2	1.00	1.26 (0.89,1.78)	0.97 (0.68,1.37)	0.86
Low HDL-C (women)				
Crude	1.00	1.25 (0.87,1.82)	0.95 (0.66,1.37)	0.81
Model 1	1.00	1.27 (0.87,1.85)	0.93 (0.64,1.34)	0.72
Model 2	1.00	1.27 (0.87,1.84)	0.92 (0.64,1.34)	0.70
Low HDL-C (men)				
Crude	1.00	1.29 (0.56,2.95)	0.73 (0.30,1.78)	0.51
Model 1	1.00	1.41 (0.60,3.29)	0.73 (0.29,1.83)	0.52
Model 2	1.00	1.50 (0.63,3.58)	0.71 (0.28,1.80)	0.50
Abdominal obesity (total)				
Crude	1.00	0.98 (0.70,1.37)	1.17 (0.83,1.63)	0.36
Model 1	1.00	0.87 (0.56,1.36)	1.17 (0.75,1.84)	0.49
Model 2	1.00	0.87 (0.56,1.36)	1.14 (0.72,1.79)	0.58
Abdominal obesity (women)				
Crude	1.00	0.97 (0.67,1.40)	1.28 (0.89,1.85)	0.19
Model 1	1.00	0.88 (0.60,1.31)	1.41 (0.96,2.08)	0.09
Model 2	1.00	0.89 (0.60,1.33)	1.36 (0.92,2.01)	0.14
Abdominal obesity (men)				
Crude	1.00	1.04 (0.40,2.75)	0.47 (0.15,1.52)	0.23
Model 1	1.00	0.93 (0.34,2.50)	0.42 (0.13,1.37)	0.16
Model 2	1.00	0.76 (0.27,2.17)	0.41 (0.12,1.42)	0.16
Metabolic syndrome (total)				
Crude	1.00	0.98 (0.68,1.40)	1.02 (0.71,1.46)	0.90
Model 1	1.00	0.89 (0.60,1.31)	1.06 (0.71,1.57)	0.78
Model 2	1.00	0.89 (0.60,1.32)	1.04 (0.70,1.54)	0.86
Metabolic syndrome (women)				
Crude	1.00	1.06 (0.72,1.56)	1.15 (0.78,1.68)	0.48
Model 1	1.00	0.99 (0.66,1.49)	1.22 (0.82,1.83)	0.33
Model 2	1.00	0.99 (0.66,1.49)	1.19 (0.79,1.78)	0.41
Metabolic syndrome (men)				
Crude	1.00	1.15 (0.46,2.90)	0.52 (0.17,1.54)	0.27
Model 1	1.00	1.20 (0.45,3.15)	0.46 (0.15,1.44)	0.20
Model 2	1.00	1.29 (0.48,3.50)	0.43 (0.14,1.39)	0.18

*Obtained by logistic regression analysis

Model 1 adjusted for age, sex (for total population), mean energy intake, smoking and physical activity, body mass index

Model 2 additionally adjusted for occupation, education and marriage

## Discussion

In this study, no significant relationship was found between MMQI and MetS and its components, except for DBP. Furthermore, stratification by sex showed that MMQI was not associated with the MetS in men or women. MMQI is one of the indexes that can be used to assess the quality of meals consumed by different populations. It helps to compare the quality of diets independently of cultural and social contexts between countries around the world (36).

To the best of our knowledge, no study has been conducted to investigate the association between MMQI and MetS. However, accumulating evidence has been studied on the relationship between the quality of a habitual diet and chronic diseases. According to a cross-sectional study by Saraf-Bank et al., performed on 1036 Iranian women, participants with a higher Healthy Eating Index (HEI) score had a 28% lower chance of developing MetS (37). Compared to the MMQI, HEI evaluates the overall diet based on 9 adequacy components and 4 moderation components that may not be adapted well for the main meals and is quite weakly correlated with MMQI. In a cohort study on 339 participants, after controlling for potential confounders, adherence to Alternative HEI (AHEI) was associated with MetS reversion, particularly in those with central obesity and those with high triglycerides (38). In another cohort study on 8719 healthy adults, HEI, Recommended Foods Score (RFS), and Dietary Diversity Score for recommended foods (DDS-R) were all strong independent negative predictors of BMI, plasma glucose, and hemoglobin A1C. The RFS and DDS-R were also inversely related to blood pressure and serum cholesterol (39). DDS-R is used to assess the diversity within food groups based on a healthy and balanced diet in various age groups that are calculated based on the consumption of different food groups including grains, meat, vegetables, fruits, and dairy products which are partially in common with MMQI (40). RFS is calculated using a minimum intake of at least half a serving of one food item from each of the cereals and white roots, green leafy vegetables, other vegetables and fruits, vitamin A-rich vegetables and fruits, organ meat, meat, fish and seafood, eggs, nut, seeds and legumes, milk, and dairy food groups (41). Unlike MMQI, DDS-R and RFS are indicators of the overall diet quality and are not meal-specific. In another cross-sectional study conducted on 300 obese Iranian adults, lower Diet Quality Index-International scores accompanied by food insecurity were associated with a higher risk of MetS, hypertriglyceridemia, reduced HDL, and increased blood pressure (42). In an article published by Felix et al. on a sample of Brazilian adults and the elderly, Breakfast Quality Index was associated with lower odds of cardiometabolic risk factors and MetS (43). The BQI was specifically developed based on the frequent foods that are eaten at breakfast to assess the nutritional quality of breakfasts in children and adolescents and is not available for other types of meals. In contrast, there was no significant association between the dietary phytochemical index (DPI) and the odds of MetS and other components of MetS in adults based on a cross-sectional study (44). DPI has many components similar to MMQI including the antioxidant-rich food groups fruits, and vegetables, but is not exclusively for meals (45). Discrepancies in the findings of studies may be related to the effect of circadian rhythm on metabolism. It seems that changes in food composition or feeding time may result in a differential response of the circadian clock.

A meta-analysis by Tian et al. showed that fruit and vegetable intakes, two components of the MMQI, were inversely associated with the risk of MetS (46). Other meta-analyses on observational studies by Zhang and Zhang, and Lee et al. confirmed the former results (47, 48). Another meta-analysis by Chen et al. found a negative association between dietary fiber intake and MetS (49). Other components of the MMQI also were influential on the prevalence of MetS per previous research (50, 51). We also did not detect any significant difference between men and women in the association of MMQI and MetS. Although sex has a significant role in determining biomarker levels of MetS and dietary behavior, sex consideration in the creation of indices related to nutrition and biomarkers is not fully studied (52). Based on the evidence, the effect of sex hormones such as progesterone, testosterone, and estrogen on appetite, energy metabolism, and eating behavior might cause a difference in the obesity prevalence of men and women (53). Furthermore, the under-representation of each sex may have an impact on the observed outcome (54).

We found no significant association between the meal-based quality index and the components of MetS. Since studies regarding meal quality and adverse outcomes are quite scarce, this study could be of greater value for future research. Recently, we reported that eating occasions and snack frequency, regardless of diet quality, increased the risk of MetS (55).

Based on the socioeconomic findings of our study, women, illiterate participants, smokers, housekeeping wives, and married individuals had a higher quality of lunch, insignificantly. In a survey of Spanish workers, being male and smoking tobacco was associated with a lower-quality of diet (56). In another study on Iranian adults, a higher quality of eating was positively associated with education, being a woman, and reversely associated with smoking and marriage (57). Although these results are insignificant, higher consumption of junk foods in restaurants as lunch might interpret the employees’ lower quality of lunch compared to housekeepers. Also. It seems that based on the existing economical gap in the society of Tehran, those who are wealthier, typically smoke more often, but in turn, have a higher meal quality.

In a study, we found that higher daily energy irregularity was linked to poorer consumption of fruits, vegetables, legumes, low-fat dairy products, and chicken, as well as higher consumption of soft drinks, processed meat, and nuts, and overall, a worse total DASH diet score and HEI-2015 (58). Furthermore, Augustina et al. in a cross-sectional survey of 335 school-going adolescent girls aged 12–19 years from Indonesia, reported an improvement in nutritional quality and diversity in a regular meal pattern by highlighting meal frequency and meal skipping (59). In another study by Gorgulho et al., it was revealed that the main meals consumed by adolescents, adults, and the elderly are not nutritionally adequate by assessing nutritional quality of the main meals, especially when consumed outdoors (23).

Meals appear to be a major driver of nutrient intake and diet quality. This could be attributed in part to the meal’s structural properties. Meals may have distinct effects on food intake, and eating patterns are complex as they are all linked to the risk factors for cardiovascular disease, and reduced nutritional intake (60). There was a negative significant association between energy, protein, total fat, saturated fat, cholesterol, polyunsaturated fat, monounsaturated fat, zinc, and sodium intakes, and MMQI scores. We also found a positive significant association between carbohydrate intake, calcium, vitamin A, and vitamin C and MMQI scores. A marginally significant relationship between vitamin E intake and MMQI was found. Previous studies have demonstrated the inverse associations of fruits, vegetables, and MetS (61, 62). High consumption of fruits and vegetables is significantly associated with a reduction in MetS (62). In this regard, this association is mediated by the high content of fiber, phytochemicals, and antioxidants in fruits and vegetables (63). Besides the well-known effects of energy density and sugars on fats on MetS, it seems that multiple bioactive substances found in each meal, such as polyphenols and fibers, act as health boosters. Polyphenols, concentrated in a large amount in vegetables and fruits that have anti-inflammatory and antioxidant properties, are a fantastic way to improve the quality of the meal. By reducing the overproduction of reactive oxygen species and suppressing free radicals, polyphenols regulate cellular and enzymatic processes involved in inflammatory pathways and play a role in glucose homeostasis as well as decreasing apoptosis and increasing pancreatic-cell proliferation, although we did not observe it in our results (64).

In the present study, the large sample size is a significant advantage, and an accurate assessment of the disorder is another strength of this study. Also, we used multiple 24-h dietary recalls. There were some limitations when interpreting the findings. The main limitation is the inability to prove causality due to the cross-sectional design of the study. Certainly, prospective cohort studies are needed to provide evidence for a causal relationship. Another concern is incorrect classification. Like other epidemiological studies, the findings of this study may not be generalizable due to the nature of the study population. Also, the 24-hour food recall may be erroneous because it is self-reported. Furthermore, because of the economic condition in Iran, most people are obligated to gain their daily protein from plant sources that are significantly cheaper than animal sources, which can cause a false increase in the MMQI score. Due to the existence of several clinical definitions of MetS, the findings may change as the MetS definition changes. It should be noted that in the present study, we used an updated definition of the Joint Scientific Statement. On the other hand, the determined waist values for abdominal obesity in Iran have been obtained from small cross-sectional studies on non-demonstration samples (65). In this study, we used international waist circumference cutoff points to ascertain central obesity. This matter might have a minor effect on the findings.

## Conclusion

MMQI is a new index designed to evaluate the quality of the main meal of the day. Since there are a few tools for healthy meal measurement, this index might add extra prospects to food choice. The findings of this study showed that the MMQI score can almost be a good predictor of the quality of the diet - MetS relationship among Iranians. Further observational and clinical studies are indeed needed to prove and cement the impact and effectiveness of MMQI on non-communicable diseases and risk factors, such as MetS.

## Data Availability

The datasets analyzed during the current study available from the corresponding author on reasonable request.

## Abbreviation:

MMQI: Main Meal Quality Index
MetS: Metabolic Syndrome
BMI: Body Mass Index
WC: Waist circumference
SBP: Systolic Blood Pressure
DBP: Diastolic Blood Pressure
LDL: Low-Density Lipoprotein
HDL: High-Density Lipoprotein
TC: Total Cholesterol
TG: Triglyceride
FPG: Fast Plasma Glucose
